# Coherence locking in a parallel nuclear magnetic resonance probe defends against gradient field spillover

**DOI:** 10.5194/mr-6-173-2025

**Published:** 2025-07-17

**Authors:** Mengjia He, Neil MacKinnon, Dominique Buyens, Burkhard Luy, Jan G. Korvink

**Affiliations:** 1 Institute of Microstructure Technology, Karlsruhe Institute of Technology, Eggenstein-Leopoldshafen, Germany; 2 Institute for Biological Interfaces 4 – Magnetic Resonance, Karlsruhe Institute of Technology, Eggenstein-Leopoldshafen, Germany; 3 Institute of Organic Chemistry, Karlsruhe Institute of Technology, Karlsruhe, Germany

## Abstract

The implementation of parallel nuclear magnetic resonance detection aims to enhance measurement throughput in support of high-throughput-screening applications, including, for example, drug discovery. In support of modern pulse sequences and solvent suppression methods, each detection site must have independent pulsed field gradient capabilities. Hereby, a challenge is introduced in which the local gradients applied in parallel detectors introduce field spillover into adjacent channels, leading to spin dephasing and, hence, to signal suppression. This study proposes a compensation scheme employing optimized pulses to achieve coherence locking during gradient pulse periods. The design of coherence-locking pulses utilizes optimal control to address gradient-induced field inhomogeneity. These pulses are applied in a pulsed-gradient spin echo (PGSE) experiment and a parallel heteronuclear single quantum coherence (HSQC) experiment, demonstrating their effectiveness in protecting the desired coherences from gradient field spillover. This compensation scheme presents a valuable solution for magnetic resonance probes equipped with parallel and independently switchable gradient coils.

## 1 Introduction

While nuclear magnetic resonance (NMR) technology is routinely employed to analyze large sets of chemical samples and to monitor dynamic biochemical processes, achieving a high-throughput capability has remained a challenge. Simultaneous detection of multiple samples enhances throughput [Bibr bib1.bibx25], while composite detection using a bundle of isolated capillaries achieves high throughput with simpler hardware [Bibr bib1.bibx27]. Greater parallel independence relies on multiple radiofrequency (RF) coils for parallel excitation and reception, along with pulsed-gradient fields [Bibr bib1.bibx25] or alternative detection [Bibr bib1.bibx23] to separate the parallel detectors. Parallel NMR has been combined with classical pulse sequences [Bibr bib1.bibx31], reaction kinetic measurements [Bibr bib1.bibx4], and dissolution dynamic nuclear polarization [Bibr bib1.bibx18]. Integrating multiple coils into a single silicon chip has demonstrated portable NMR applications [Bibr bib1.bibx21], employing time-interleaved pulses for RF decoupling. For full parallel and independent operation, each detector typically integrates individual RF coils, gradient coils, and shimming units [Bibr bib1.bibx3]. However, practical limitations in electromagnetic shielding design, particularly in dense and highly composite arrays, result in field leakage and inter-channel coupling. RF decoupling schemes have been reported recently with regard to both the excitation and reception stages [Bibr bib1.bibx12]. The pulsed-gradient field spillover among parallel detectors directly induces $B_{0}$ inhomogeneity, leading to spin dephasing, thus posing a challenge that remains to be addressed.

A straightforward approach to mitigate spin dephasing to $B_{0}$ inhomogeneity is by transferring the spin state to longitudinal magnetization, e.g., $I_{z}$ for single spins or $I_{z}S_{z}$ for coupled spins. However, this proves to be challenging when the initial state is unknown or when time constraints exist. Spin locking via RF pulses is an effective strategy for preserving spin magnetization; i.e., a long hard pulse with a defined phase is applied to protect a specific coherence, such as $I_{x}$ or $I_{y}$ . In heteronuclear experiments, continuous-wave spin-locking fields have been utilized to render proton multiple quantum coherence immune to ^1^H–^1^H J coupling [Bibr bib1.bibx11]. Spin-locking-induced crossing has a wide range of applications, including preparation of singlet states [Bibr bib1.bibx6], excitation of long-lived states [Bibr bib1.bibx28], and low field spectroscopy [Bibr bib1.bibx9]. Moreover, traditional spin-locking pulses have been adapted to address $B_{0}$ and $B_{1}$ inhomogeneity in quantifying the rotating frame relaxation time [Bibr bib1.bibx17].

In this study, we used optimal control to design cyclic pulses that preserve the desired coherence, such as $I^{-}$ for a single spin and $I^{-}S^{+}$ for a coupled spin pair. The coherence locking by optimal control (CLOC) pulses exhibit robustness against a range of $B_{0}$ drifts, effectively mitigating gradient spillover effects in parallel NMR experiments. Multiple CLOC blocks are inserted to safeguard specific coherence transfer pathways when the pulse sequence incorporates a series of gradient pulses. Using this protection idea, we demonstrated that the parallel HSQC experiment retains its signal-to-noise ratio (SNR) through coherence locking.

## 2 Results and discussion

Figure [Fig F1]a shows the geometry of the parallel detector used in the experiments. Each detector is equipped with a Helmholtz gradient coil and a strip line RF coil. To provide an initial view, three scenarios were considered for the HSQC experiments. The first scenario, serving as a reference, is shown in the black part of Fig. [Fig F1]b. A standard HSQC pulse sequence was used, in which gradient pulses with a ratio of 2:2:-1 were applied to select the $S^{+}\to S^{+}\to I^{+}\to I^{-}$ coherence transfer pathway. The second scenario, depicted in orange in Fig. [Fig F1]b, introduces an additional set of gradient pulses $G_{C}$ . These gradients, independent of G , were applied with a ratio of 2:2:1 and were temporally shifted relative to the primary gradients to induce gradient field spillover. The third scenario, indicated in blue in Fig. [Fig F1]b, integrates three CLOC blocks along with $G_{C}$ into the HSQC sequence. The first and second CLOC blocks were applied to ^13^C, while the third was applied to ^1^H.

**Figure 1 F1:**
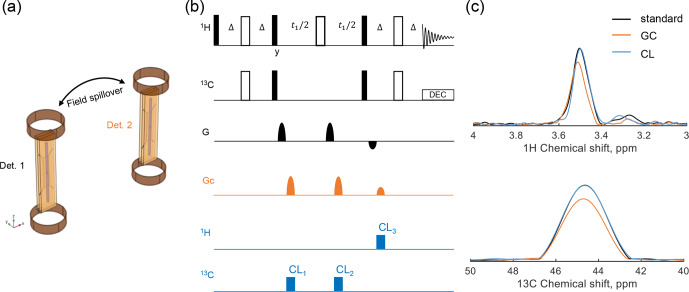
Setup of the HSQC experiment under gradient field spillover. **(a)** The parallel detector geometry, where each detector is equipped with a Helmholtz gradient coil and a strip line. **(b)** Pulse sequences of the three experimental HSQC scenarios. The black part represents a standard HSQC pulse sequence, with a gradient pulse ratio of 2:2:-1 to select the $S^{+}\to S^{+}\to I^{+}\to I^{-}$ coherence pathway, defining the first scenario. The orange part ( $G_{C}$ ) represents additional gradient pulses from a second detector, set at a 2:2:1 ratio, inserted into HSQC as coupled gradients, defining the second scenario. The blue section indicates the insertion of CLOC pulses into HSQC, aligned with the coupled gradient pulses, representing the third scenario. Specifically, ${\mathrm{CL}}_{1}$ and ${\mathrm{CL}}_{2}$ are applied to ${}^{13}C$ , while ${\mathrm{CL}}_{3}$ is applied to ${}^{1}H$ . **(c)** The 1D projections of HSQC spectra corresponding to the three experimental scenarios, obtained using 0.6 M glycine-2-^13^C in $D_{2}O$ .

Experiments were conducted for all three scenarios using glycine-2-^13^C as the test sample, and the resulting spectra are shown in Fig. [Fig F1]c. Compared to the reference spectrum from the standard HSQC experiment, the gradient-coupling scenario resulted in a 20 % signal loss. In HSQC, a coherence pathway is refocused only if the net effect of all gradient pulses is zero. Additional gradients can disrupt this balance, causing dephasing and signal loss, which can happen when separate gradient-enhanced experiments are run in parallel at two detector sites. However, including CLOC blocks resulted in a signal intensity equal to the reference, indicating that the relevant coherences were preserved. Different gradient ratios are utilized in a parallel probe if multiple detectors execute different experiments or employ the same sequence but select distinct coherence pathways. The gradient spillover disrupts the intended coherence, and coherence locking can mitigate the dephasing effect in these scenarios. Note that, if two detectors use identical sequences with the same gradient pulse ratio, the total gradient ratio remains unchanged. Consequently, no gradient defense mechanisms are required, as shown in Fig. S2b.

Following the overview, we detail the gradient-coupling issue and the coherence-locking compensation solution. The simulation results quantifying the gradient spillover effect are presented in Sect. S1 in the Supplement, and the measurement of the gradient spillover ratio is provided below. Firstly, the maximum gradient strength of the parallel probe was measured using the pulsed-gradient spin echo (PGSE) experiment for a sample of $10\;\%\;H_{2}O/90\;\%\;D_{2}O$ with a diffusion constant of $D=1.9\times 10^{-9}\;m^{2}\;s^{-1}$ [Bibr bib1.bibx15]. The ratio of the signal to the maximum value is a function of the applied gradient amplitude [Bibr bib1.bibx29]:

1 $$\ln(I_{g}/I_{0})=-[\gamma^{2}\delta^{2}G^{2}(\Delta -\delta )]D,$$

where δ=1ms is the gradient pulse length, Δ=7ms is the interval between two gradient pulses, and $\gamma =2.675\times 10^{8}\;\mathrm{rad}\;s^{-1}\;T^{-1}$ is the proton gyromagnetic ratio. Curve fitting yielded a maximum gradient of $G_{\max}=103.06\;G\;{\mathrm{cm}}^{-1}$ ; see Fig. [Fig F2]a.

**Figure 2 F2:**
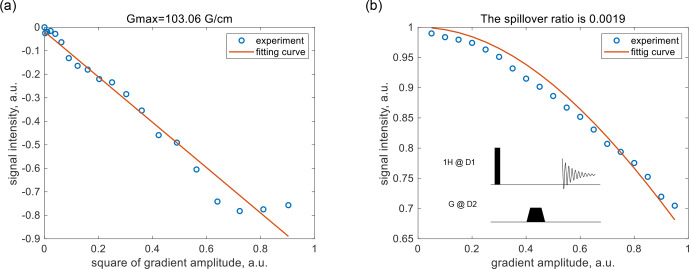
Measurement of the gradient spillover ratio for the parallel detector in Fig. [Fig F1] using $10\;\%\;H_{2}O/90\;\%\;D_{2}O$ . **(a)** Signal intensity as a function of gradient amplitude in the PGSE sequence; the maximum gradient amplitude was calculated to be $G_{\max}=103.06\;G\;{\mathrm{cm}}^{-1}$ at a 100% gradient. **(b)** Signal intensity as a function of coupling-gradient amplitude in a pulse acquisition experiment; the gradient spillover ratio was calculated to be $R_{G}=1.9\times 10^{-3}$ . The circles and the solid lines represent the experimental data and the fitted curves, respectively.

The gradient spillover ratio was then measured in a pulse acquisition experiment, while a gradient pulse was applied to the second detector; see the insert in Fig. [Fig F2]b. The ratio of the signal to the maximum value is a function of the applied gradient amplitude:

2 $$\frac{I_{g}}{I_{0}}=\frac{\sin(\text{kg})}{\text{kg}},$$

where $k=l/2\cdot \gamma \cdot R_{G}\cdot G_{\max}\cdot \int g(t)\;dt$ , l=6.5 mm is the detection zone length, and ∫g(t)dt=0.9 ms is the time integral of the trapezoidal gradient pulse. The gradient spillover ratio was determined by curve fitting to be $R_{G}=1.9\times 10^{-3}$ .

Depending on the gradient pulses used, the impact of such a gradient spillover can differ. For example, the gradient coupling resulted in 20% signal loss in an HSQC experiment, as shown in Fig. [Fig F1]c. Strong gradients are essential for diffusion experiments as they can induce a coupled gradient of 0.20 $G\;{\mathrm{cm}}^{-1}$ in the neighboring detector, making gradient coupling a critical concern. Here, we propose a compensation scheme utilizing RF pulses to mitigate the gradient spillover. The idea is to protect desired coherences from unwanted field gradients using CLOC pulses, which are time-aligned with the coupling-gradient pulses. For example, a gradient pulse applied to detector 1 can be compensated for by simultaneously applying a CLOC pulse to detector 2. This scheme was demonstrated with a PGSE experiment and a parallel HSQC experiment.

**Figure 3 F3:**
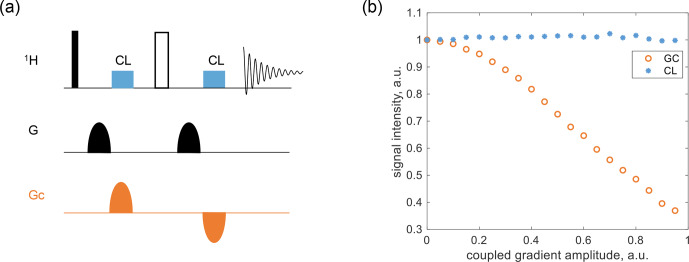
Coherence-locking test in PGSE using 0.6 M glycine-2-^13^C in $D_{2}O$ . **(a)** The pulse sequence, where the ^1^H pulse and G were applied to detector 1, and $G_{C}$ was applied to detector 2. **(b)** The signal intensity of the water peak, normalized to its value when $G_{C}=0$ , was plotted as a function of $G_{C}$ , with G fixed at 10% .

As shown in Fig. [Fig F3]a, an additional gradient, $G_{C}$ , was introduced as the coupling component in a standard PGSE sequence, temporally shifted relative to the primary gradient. When $G_{C}$ disrupted the strength balance on either side of the inversion pulse, two CLOC pulses were applied, aligned with each block of $G_{C}$ , to counteract gradient spillover. These CLOC pulses preserved spin coherence, protecting $I^{+}$ during the first $G_{C}$ period and $I^{-}$ during the second. Alternatively, the pulses can enable effective cyclic propagation (i.e., U=1 ), which is a stricter condition and was selected as our design target. Details of pulse optimization are provided later. The proposed coherence locking in PGSE was tested using 0.6 M glycine-2-^13^C in $D_{2}O$ , with results shown in Fig. [Fig F3]b. The $G_{C}$ amplitude was swept from 0 % to 95% in the pulse program, while G was fixed at 10% , and the water peak intensity was extracted and normalized to its value at $G_{C}=0$ for comparison. Compared to the large signal loss in the gradient-coupling case, the CLOC pulse effectively restored the signal intensity.

**Figure 4 F4:**
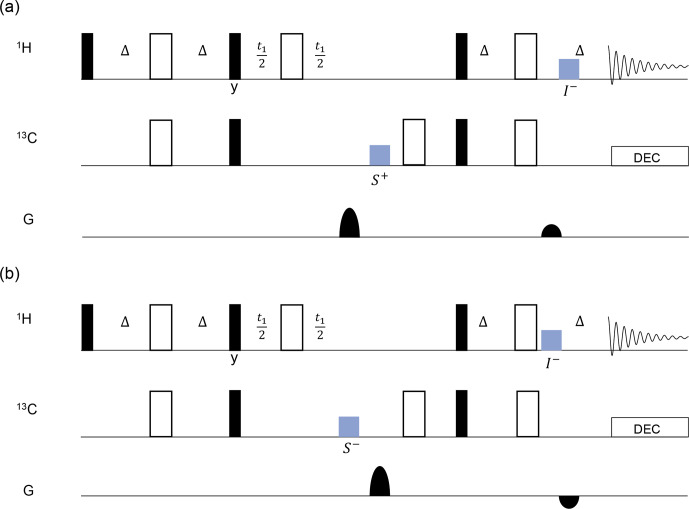
The scheme for gradient pulse compensation in a parallel HSQC pulse sequence involves blocks (colored in blue) indicating the CLOC pulse. The gradient pulse ratio in detector 1 **(a)** was set to 4:1 to select the $S^{+}\to I^{-}$ pathway. In detector 2 **(b)**, the gradient pulse ratio was set to 4:-1 to select the $S^{-}\to I^{-}$ pathway. The black blocks represent π/2 pulses, and the white blocks represent π pulses, with all phases set to 0 unless specifically noted. Δ=1/4J . The protected coherences are labeled below the CLOC blocks.

A pulse sequence tailored for parallel HSQC is illustrated in Fig. [Fig F4], in which the fundamental HSQC sequences were implemented in detectors 1 and 2. The gradient ratio in detector 1 was set to 4:1 to select the $S^{+}\to I^{-}$ pathway (Fig. [Fig F4]a), while, in detector 2, it was adjusted to 4:-1 to select the $S^{-}\to I^{-}$ pathway (Fig. [Fig F4]b). The compensation CLOC pulses, indicated in blue, were applied in detector 1, while the gradient pulses were applied in detector 2 and vice versa. The gradient pulses in parallel detectors were executed with a slight time delay to allow for the insertion of the CLOC pulses. Each compensation pulse was applied to lock onto the relevant coherence. For instance, the first CLOC block in detector 1 was applied on ^13^C to protect $S^{+}$ and its J coupling-induced product $I_{z}S^{+}$ . The protected coherences are labeled below the CLOC pulses in Fig. [Fig F4]. With this approach, the CLOC pulse must be designed to compensate for gradient spillover (effectively a range of frequency offsets) to protect one or more desired coherences.

In the pulse optimization, both the source and target states were specified as the locked coherence. When a gradient pulse is applied, its shape and duration remain fixed, while its amplitude can vary. The optimization of CLOC pulses adheres to this principle. Specifically, the gradient pulse contributes to a time-dependent drift Hamiltonian $H_{g}(t)=A\sin(\pi t/\tau )H_{z0}$ , where $H_{z0}$ represents the Zeeman Hamiltonian under a 1 T field, and τ=1 ms is the gradient pulse duration. To cover a maximum $B_{0}$ drift of ±0.06 Gauss , measured with the parallel probe, the maximum $B_{0}$ drift of ±0.25 Gauss was specified for the CLOC pulse. Therefore, multiple time-dependent drifts were included in the optimization to account for both spatial and temporal variations in $B_{0}$ drifts. The RF amplitude in the ^1^H channel was set to 6 kHz , with a $\pm 20\;\%\;B_{1}$ inhomogeneity, covering a 7 kHz bandwidth and, simultaneously, a maximum $B_{0}$ drift of ±0.25 Gauss , corresponding to ±1.07 kHz offset. For the ^13^C channel, the RF amplitude was adjusted to 4 kHz , with a $\pm 15\;\%\;B_{1}$ inhomogeneity, covering a 6 kHz bandwidth and, simultaneously, a maximum $B_{0}$ drift of ±0.25 Gauss . Decoupling of heteronuclear J coupling is demonstrated below, and the compensation for homonuclear J coupling is discussed in Sect. S4 in the Supplement. Although concurrent optimization can explicitly include the J -coupling term in the system Hamiltonian, it involves parameters from two spins, resulting in a large model. Therefore, the strategy is to build optimal control for a single spin and test its heteronuclear decoupling effect within the designed bandwidth. The decoupling effect was quantified using average Hamiltonian theory [Bibr bib1.bibx32]. The J -coupling scale factor is given by the following:

3 $$\chi =\mathrm{norm}(\bar{c}),$$

where c is the time-dependent J -coupling tensor in the toggling frame, defined by the RF pulse plus resonance offset (see Sect. S4). Figure [Fig F5] displays the χ values for CLOC pulses designed for universal locking of $I^{+}$ , $I^{-}$ , and $I_{z}$ of a single spin. A single CLOC pulse reduces heteronuclear J coupling by less than 10% across the designed resonance offset and $B_{1}$ inhomogeneity range; see Fig. [Fig F5]a and b. Given a pulse duration of 1 ms, heteronuclear J couplings smaller than or on the order of hundreds of hertz can be ignored as χJτ≪1 . This treatment also applies to coupling with a third spin when studying ^1^H and ^13^C, such as $J_{\mathrm{CN}}$ and $J_{\mathrm{HN}}$ in proteins [Bibr bib1.bibx22]. Figure [Fig F5]c shows that residual coupling exists when two CLOC pulses are applied simultaneously to ^1^H and ^13^C. However, it does not imply that the decoupling fails as χ sums all the items of the heteronuclear J -coupling tensor. One can also calculate the coherence-locking efficiency when double-quantum coherences require protection. The coherence locking for double-quantum coherence was tested by simulating a heteronuclear multiple quantum coherence (HMQC) sequence, as detailed in Sect. S7.

**Figure 5 F5:**
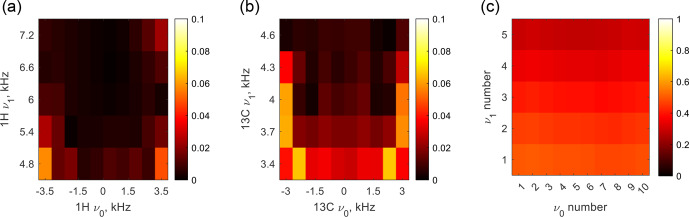
Scale factor χ of the $J_{\mathrm{HC}}$ coupling as a function of resonance offset and $B_{1}$ amplitude. **(a)** The CLOC pulse is applied to ^1^H, while ^13^C is on resonance. **(b)** The CLOC pulse is applied to ^13^C, while ^1^H is on resonance. **(c)** The CLOC pulses are applied simultaneously to ^1^H and ^13^C, with $\nu_{0}$ and $\nu_{1}$ aligned on both channels.

Using this strategy, the simulated locking efficiency of the optimal control pulses is depicted in Fig. [Fig F6]. The sample was segmented into 12 voxels along the z direction, and the spin trajectory for each voxel was computed while simultaneously executing a CLOC pulse and a coupled gradient pulse, which induced a maximum $B_{0}$ drift of ±0.25 Gauss . Figure [Fig F6]a illustrates that the ensemble spin states start from $I^{-}$ and oscillate between -1 and 1 while remaining confined within a narrow range and then go back to $I^{-}$ at the end of a 1 ms gradient pulse. Figure [Fig F6]b and c show the evolution of ensemble spin states starting from $S^{+}$ and $I^{-}S^{+}$ , respectively. Additionally, Fig. [Fig F6]d–f present the spin trajectory with the same initial states but without coherence locking for comparison, indicating coherence dephasing caused by the coupled gradient.

**Figure 6 F6:**
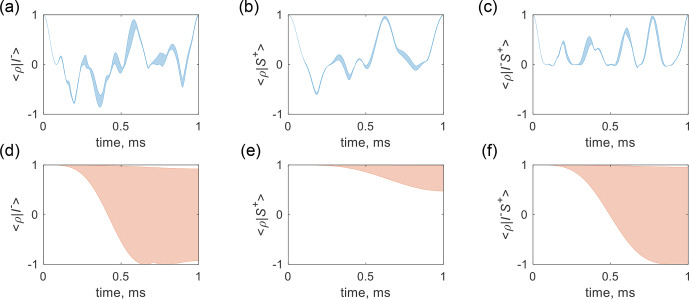
Ensemble spin trajectory subject to the gradient field spillover dephasing. Panels **(a)**, **(b)**, and **(c)** represent the trajectories starting from $I^{-}$ , $S^{+}$ , and $I^{-}S^{+}$ , with the CLOC pulses applied to ^1^H, ^13^C, and ^1^H & ^13^C, respectively. Panels **(d)**, **(e)**, and **(f)** correspond to the respective trajectories without CLOC pulses. The real values of the inner product are displayed. The ^1^H and ^13^C were on resonance, and the J coupling constant was 145 Hz . The RF amplitudes were 6 kHz for ^1^H and 4 kHz for ^13^C.

**Figure 7 F7:**
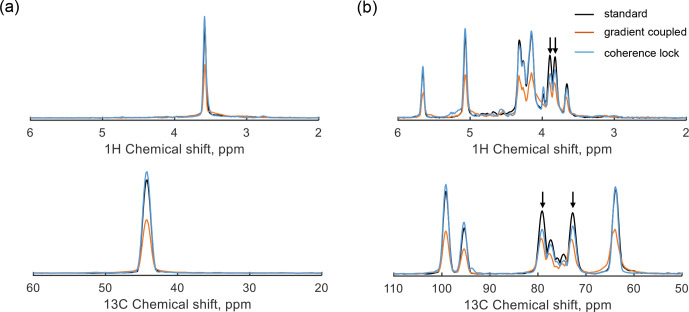
Experimental parallel HSQC spectra of glycine-2-^13^C **(a)** and D-glucose- ${}^{13}C_{6}$ **(b)** using the pulse sequence in Fig. [Fig F4]. The gray lines represent the results without gradient coupling as a reference. The orange lines display the spectrum under gradient spillover (no compensation), and the blue lines display the results with coherence-locking compensation.

The parallel HSQC pulse sequence was tested using a parallel probe (see Sect. [Sec Ch1.S3] for details); two detectors of the probe were used, with 0.6 M glycine-2-^13^C in $D_{2}O$ in detector 1 and 0.3 M D-glucose- ${}^{13}C_{6}$ in $D_{2}O$ in detector 2. The resulting spectra are presented in Fig. [Fig F7], where the 1D projections of the ^1^H and ^13^C signals from the 2D spectrum are displayed to illustrate the signal intensity for three cases. The single-detector scenario (gray) serves as a reference, while the orange lines represent amplitude-suppressed signals caused by gradient spillover-induced dephasing. In contrast, the blue lines correspond to the results of parallel operation with coherence locking, demonstrating signal intensity recovery and effective dephasing compensation. Suppose the CLOC pulses effectively achieve broadband locking for both the ^1^H and ^13^C spin coherences, such that each peak in the coherence-locking case has the same intensity as in the reference case. However, in Fig. [Fig F7]b, slight intensity differences are observed between the coherence-locking results and the reference, particularly for the peaks at 3.8 and 3.9 ppm in the ^1^H dimension, correlating to 72.8 and 79.1 ppm in the ^13^C dimension, respectively. Since the pulse optimization did not compensate for homonuclear coupling (^1^H–^1^H and ^13^C–^13^C), coherence-locking efficiency is degraded, especially when the offset difference between two peaks is comparable to the homonuclear J -coupling constant. A comparison of D-glucose- ${}^{13}C_{6}$ HSQC spectra with and without homonuclear coupling is provided in Sect. S6. The discrepancies also arise from the chemical shift dependence of coherence-locking efficiency. For instance, each CLOC pulse introduces slight phase distortion; if each pulse has a fidelity of 95% , the combined fidelity of two pulses is reduced to 90% . The robustness of CLOC pulses encompasses factors including bandwidth, $B_{1}$ inhomogeneity, and gradient amplitude, which are detailed in Sect. S5. In addition, coherence locking can also be influenced by RF coupling; when a CLOC pulse is applied in detector 1, weak RF signals (about 1% at 500 MHz [Bibr bib1.bibx12]) may be transferred from detector 1 to detector 2, distorting the spin state. Hence, the CLOC pulse was adjusted to relatively lower amplitudes, i.e., 6 kHz for ^1^H and 4 kHz for ^13^C. The corresponding power levels were 0.43 W for the ^1^H channel, and 2.3 W for the ^13^C channel on detector 2, making the RF coupling effect more significant in the ^13^C channel, highlighting the challenges of coherence locking for low-sensitivity nuclei when a large bandwidth is required.

## 3 Methods

For sample preparation, a $10\;\%\;H_{2}O/90\;\%\;D_{2}O$ solution was prepared to measure the gradient strength and gradient spillover ratio. Two solutions were prepared in $D_{2}O$ ( 99.9% ): a 0.6 M glycine-2-^13^C solution and a 0.3 M D-glucose- ${}^{13}C_{6}$ solution for the parallel HSQC experiment. The same glycine-2-^13^C solution was also used for the PGSE experiment. The samples were loaded into syringes and manually pumped into the individual fluidic chambers of the dedicated detector. The $D_{2}O$ , glycine-2-^13^C, and D-glucose- ${}^{13}C_{6}$ were purchased from Sigma-Aldrich.

Experimental validation was performed using a four-detector parallel NMR probe (Voxalytic GmbH), as shown in Fig. S12, which was installed in a Bruker AVANCE NEO 11.7 T (^1^H frequency of 500.13 MHz) NMR system (Bruker BioSpin GmbH). For demonstration, two of the four detectors were used, each double-resonant ( ${}^{1}H{/}^{13}C$ ) and equipped with an independent single-axis pulsed field gradient. The two gradient channels were powered by the Bruker GREAT micro-imaging amplifiers using a customized cable splitting the three amplifiers from a single cable into three individual cables (Bruker). Pulse calibration was conducted for each detector individually. In detector 1, a hard 90° ^1^H of pulse length 7.4 ms was applied at 20 W , and a hard 90° ^13^C pulse of length 25 ms was applied at 25 W , with a corresponding value in detector 2 of 6.1 ms for ^1^H and of 19 ms for ^13^C. The power levels were then scaled down for the CLOC pulses with low amplitudes. The HSQC contained 256 $t_{1}$ increments, each with one scan of 1024 data points. A total of 32 dummy scans were executed to stabilize the spin system before data collection; the relaxation delay was 1 s, and the receiver gain is 10 . The sweep width was 10 ppm for ^1^H and 150 ppm for ^13^C. Experiments explicitly run in parallel were done using the multi-receive option in TopSpin 4.1.3. The parallel HSQC experiments were repeated twice for good shimming quality: data from detector 1 were collected with global shimming focused on detector 1, and data from detector 2 were collected with shimming focused on detector 2.

The magnetic-field simulation was conducted with the finite-element method software COMSOL MultiPhysics 6.1 [Bibr bib1.bibx5], and the simulated data were processed with MATLAB (2023b) [Bibr bib1.bibx30]. The pulse optimization and spin dynamic calculations were completed with Spinach v2.8 [Bibr bib1.bibx14]; the detailed setting for optimal control is provided in Sect. S3.

## 4 Conclusion

We employed CLOC pulses to protect specific coherences from gradient spillover in a parallel NMR setup. This approach effectively compensates for gradient-induced phase shifts, preserving coherence and signal integrity across parallel detectors, as demonstrated in a parallel HSQC experiment. This optimal control-assisted coherence locking provides an alternative strategy for designing coherence protection protocols.

Although we tested the heteronuclear decoupling effect of specific optimal pulses, general optimal pulses could potentially average out the heteronuclear coupling due to their “noise-like” waveforms. The overall strategy is to establish the locking of a single spin and validate its decoupling, thereby avoiding the complexity of a coupled spin model. While the CLOC pulses protect the targeted coherences, allowing coherence evolution to be neglected during this period, it should be excluded when calculating the J -coupling evolution delay.

The limitations of this approach can stem from RF coupling between multiple detectors and homonuclear coupling in a general spin system. As shown by a parallel HSQC experiment and HMQC simulations, single-spin coherence can be universally locked in a general spin system, while double-quantum coherences ( IS ) can be locked using two simultaneous locking pulses. However, locking higher-order heteronuclear coherence is challenging in practice as multiple coherence-locking pulses can introduce significant RF coupling. Although optimal control can compensate for homonuclear coupling at the cost of increased RF power, the compensation fails when two spins have a small chemical shift difference and exhibit second-order spectra, where the RF Hamiltonian commutes with the J -coupling term, and the Zeeman term nearly commutes with the J -coupling term. Both high-order coherence locking and homonuclear decoupling require additional RF power; a broader approach may jointly compensate for RF coupling and gradient spillover effects. The limitation of extending this scheme to higher parallelism also arises from relaxation decay, which increases with more alternating gradient pulses.

This work discussed conducting the same pulse sequence in two detectors with different gradient ratios. When the detectors perform different experiments, such as HMQC on detector 1 and HSQC on detector 2, the relaxation delay on each detector can be adjusted to synchronize the parallel pulses. However, because the acquisition is no longer simultaneous, addressing the RF coupling between the pulse and free induction decay in the acquisition stage is beyond the scope of this work.

## Supplement

10.5194/mr-6-173-2025-supplement The supplement related to this article is available online at https://doi.org/10.5194/mr-6-173-2025-supplement.

## Supplement

10.5194/mr-6-173-2025-supplement
10.5194/mr-6-173-2025-supplement The supplement related to this article is available online at https://doi.org/10.5194/mr-6-173-2025-supplement.

## Data Availability

The MATLAB code and experimental data are available at 10.5281/zenodo.15188023 [Bibr bib1.bibx13].

## Appendix

The supplement related to this article is available online at https://doi.org/10.5194/mr-6-173-2025-supplement.
