# Supplementary material for: Coherence locking in a parallel nuclear magnetic resonance probe defends against gradient field spillover
Source: Magn Reson (Gott). 2025 Jul 17;6(2):173–81. doi: 10.5194/mr-6-173-2025 (PMC12302023; doi:10.5194/mr-6-173-2025)
Supplement: The supplement related to this article is available online at https://doi.org/10.5194/mr-6-173-2025-supplement. [file mr-6-173-2025-supplement.pdf]

Supplement of Magn. Reson., 6, 173–181, 2025
https://doi.org/10.5194/mr-6-173-2025-supplement
© Author(s) 2025. CC BY 4.0 License.

Supplement of

Coherence locking in a parallel nuclear magnetic resonance probe defends against gradient field spillover

Mengjia He et al.

Correspondence to: Neil MacKinnon (neil.mackinnon@kit.edu) and Jan G. Korvink (jan.korvink@kit.edu)

The copyright of individual parts of the supplement might differ from the article licence.

Contents

S1. Two-detectror array gradient simulation 2

S2. Dephasing by gradient field spillover 4

S3. Optimization of coherence-locking pulse 6

S4. Heteronuclear decoupling and homonuclear *J*-coupling impact 9

S5. Simulation of coherence-locking efficiency 11

S6. Simulation of parallel HSQC 12

S7. Simulation of parallel HMQC 15

S8. Influence of gradient imperfection 18

S9. The parallel probe and parallel HSQC spectra 19

S1. Two-detectror array gradient simulation

The gradient current in detector 1 was represented by $i_1(t) = 3\sin(1000\pi t)$, as shown in Fig. S1b. This current generates a field gradient of 75 Gauss/cm in sample 1, although not explicitly shown. Two situations now arise at detector 2: first, the applied gradient at detector 1 can induce a current in the coil at detector 2, thereby creating an opposing field. Second, the gradient field generated at detector 1 is not fully shielded, so that this field spillover penetrates the sample at detector 2. The induced current i_2 in detector 2 due to the magnetic flux change was computed in Fig. S1d. Meanwhile, Fig. S1c portrays the magnetic field in sample 2 generated by i_1 and i_2. Notably, the gradient amplitude at detector 2 from i_1 amounted to 0.5 Gauss/cm, resulting in a spillover ratio of 0.67%. This value is approximately 68 times higher than the maximum gradient generated from the induced current i_2, echoing similarities with Lenz's law, where the induced current's magnetic flux opposes the change in flux but cannot fully counteract it. Consequently, the field generated by the induced current was disregarded in subsequent analyses. Fig. S1c highlights that field spillover induces an additional gradient contribution alongside the primary gradient. When multiple gradient pulses are applied to select a coherence transfer pathway, the designed gradient ratio may undergo distortion due to field spillover.

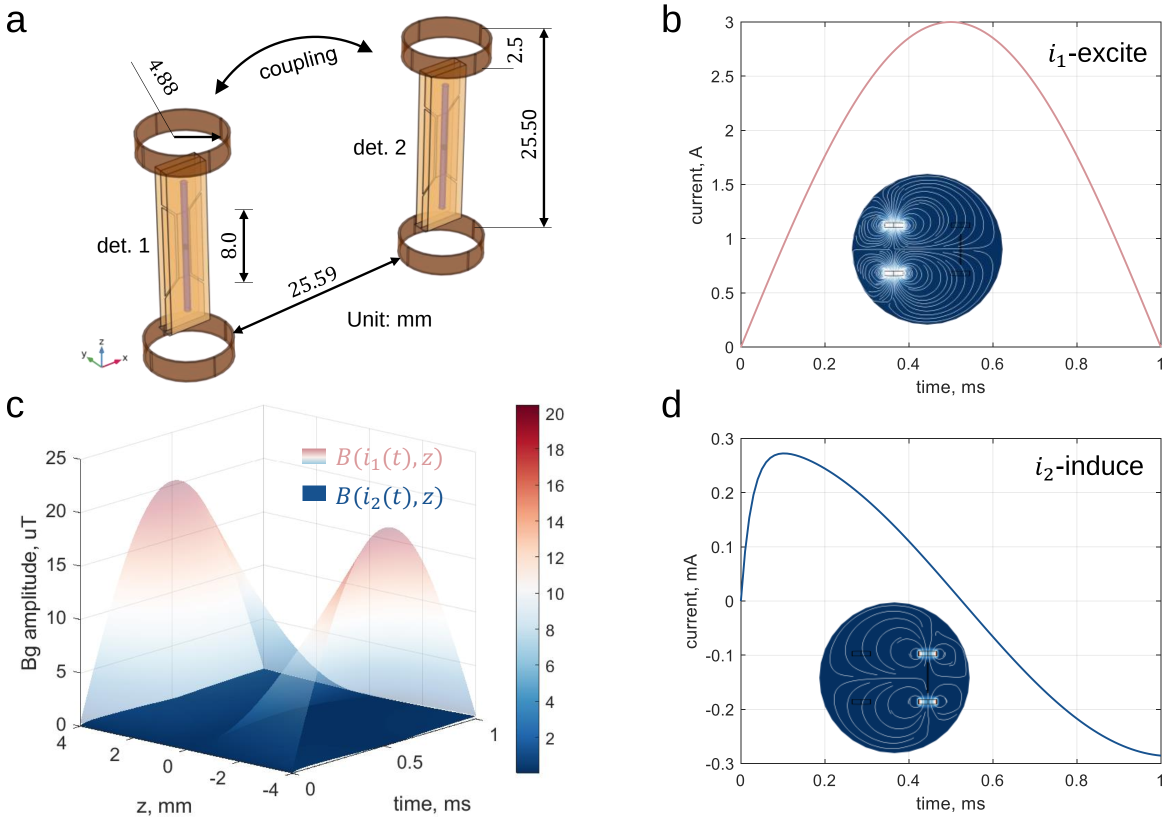

Figure S1. Gradient simulation for a two detector array. (a) Geometry of the detector array. (b) The gradient coil in detector 1 was excited with a sin-shaped pulse. (c) The gradient field on sample 2 calculated with the excited current $i_1(t)$ and induced current $i_2(t)$, the ratio of maximum $B(i_1)$ and $B(i_2)$ is 68. (d) Induced current $i_2(t)$ in detector 2.

S2. Dephasing by gradient field spillover

For the evolution of p quantum coherence under the influence of a field gradient pulse $G_z(t)$, with duration τ, the spin coherence after the gradient pulse is given by[1]

$$\rho(\tau) = \rho(0)\exp[-ip\int_0^{\tau} \gamma G_z(t) z\,dt] \tag{S1}$$

where z is the spatial coordinate, and γ is the gyromagnetic ratio. The phase of the coherence after the pulse is

$$\phi_p(z) = -p\int_0^{\tau} \gamma G_z(t) z\,dt \tag{S2}$$

which forms a helix concerning the spatial coordinate. The wavelength of the helix is given by

$$\lambda = -2\pi/[p\int_0^{\tau} \gamma G_z(t)\,dt] \tag{S3}$$

So $\phi_p(z)$ can be also expressed as $\phi_p(z) = 2\pi z/\lambda$

For a coherence transfer pathway with N coherences $[p_1, p_2, ...p_N]$, Eq. S2 is expanded to

$$\phi_{p_1\to p_N}(z) = -\sum_{i=1}^{N} p_i \int_{\tau_i} \gamma_i G_z(t) z\,dt \tag{S4}$$

And Eq. S3 is accordingly expanded to

$$\lambda_{p_1\to p_N} = -2\pi/[\sum_{i=1}^{N} p_i \int_{\tau_i} \gamma_i G_z(t)\,dt] \tag{S5}$$

To determine the decay in signal intensity induced by spillover, we consider a sample of length l. The signal intensity is proportional to integrating the phase term across the entire sample, expressed as

$$s = \int_{-l/2}^{l/2} e^{i\phi(z)}, dz = \int_{-l/2}^{l/2} e^{i2\pi z/\lambda}, dz = \frac{\lambda}{\pi}\sin\left(\frac{\pi l}{\lambda}\right) \tag{S6}$$

where λ represents the wavelength of the gradient-induced phase helix corresponding to the coherence transfer pathway, see Eq. S5. When λ approaches infinity, the signal achieves its maximum amplitude $s_{\max} = l$, effectively extracting the specified coherence transfer pathway. However, when λ is not sufficiently large or comparable to the sample size, the signal intensity is given by

$$\frac{s}{s_{\max}} = \frac{\lambda}{\pi l}\sin\left(\frac{\pi l}{\lambda}\right) \tag{S7}$$

which follows the well-known sinc function, as depicted in Fig. S2a. The effect on signal intensity in the case of a parallel NMR measurement is plotted in Fig. S2b. The signal intensities were extracted from a simulated parallel ^{1}H-^{13}C HSQC experiment, employing the fundamental HSQC pulse sequence[2], using the detector geometry outlined in Fig. S1. Each detector was subjected to 'sin' shaped gradients with a duration of 1 ms, considering spillover ratios which is defined as the ratio between the maximum coupled gradient strength (G_{couple}) and the maximum primary gradient strength (G_{primary}), expressed as $R_G = G_{\text{couple}}/G_{\text{primary}}$, ranging from 0.1% to 1%. The gradient ratio in detector 2 was fixed at 2:2:-1, while for detector 1, the gradient ratios of 2:2:1 (blue) and 2:2:-1 (orange) were considered.

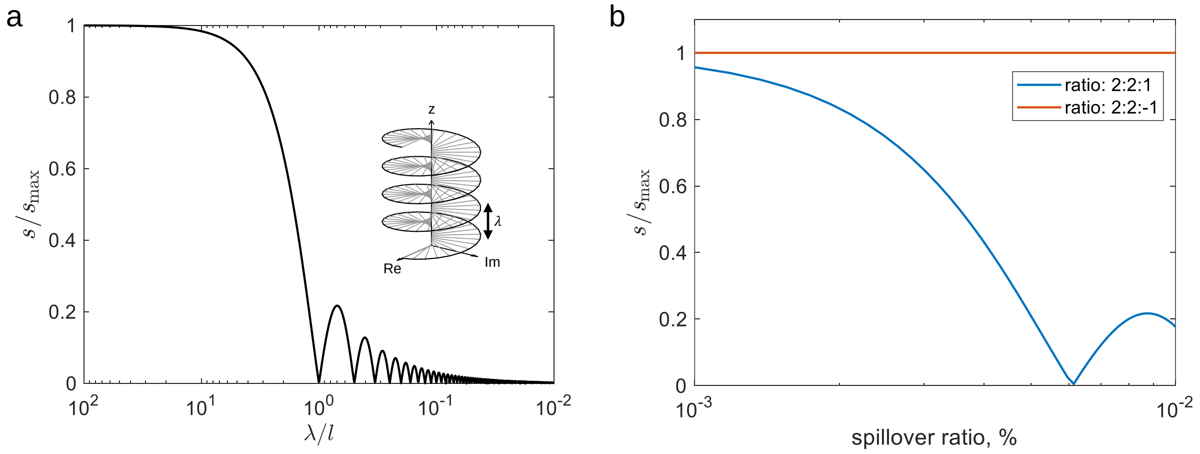

Figure S2. Signal intensity under field gradient dephasing. (a) Relative signal intensity plotted against helix wavelength. (b) Relative intensity of the parallel HSQC signal depicted against gradient spillover ratio. The gradient ratio in detector 2 is 2:2:-1, and the gradient ratio in detector 1 is 2:2:1 (blue) and 2:2:-1 (orange) respectively. The primary gradient strength is fixed at 75 Gauss/cm which is reached at the first and second gradient pulse, the sample length is 8 mm, and the x-axis is presented on a logarithmic scale.

S3. Optimization of coherence-locking pulse

Optimal control theory Here, we provide a spin dynamics description of the optimal control for coherence locking, within the Liouvillian space. In this space, the Hamiltonian is represented as the commutation superoperator, and the spin state is represented as a column vector. Without loss of generality, we consider coherence locking of a two-spin system, i.e., I and S. The rotating frame Hamiltonian of the spin system can be written as the internal part plus the control parts as

$$\mathbf{H}(t) = \mathbf{H}_{\text{int}} + \mathbf{H}_{\text{rf}}(t) + \mathbf{H}_{\text{g}}(t) \tag{S8}$$

$$\mathbf{H}_{\text{int}} = \omega_I I_z + \omega_S S_z + 2\pi J I_z S_z \tag{S9}$$

$$\mathbf{H}_{\text{rf}}(t) = \sum_i \omega_{i,x}(t) I_{i,x} + \omega_{i,y}(t) I_{i,y} \tag{S10}$$

$$\mathbf{H}_{\text{g}}(t) = B_{\text{g}}(t) \cdot \mathbf{H}_{\text{z0}} \tag{S11}$$

where $\mathbf{H}_{\text{z0}}$ is the labframe Zeeman Hamiltonian within 1 T magnetic field, the $B_{\text{g}}(t)$ indicates a time-dependent field drift by the gradient, so $\mathbf{H}_{\text{int}} + \mathbf{H}_{\text{g}}(t)$ represents a time-dependent drift Hamiltonian. The $\omega_{i,x}(t)$ and $\omega_{i,y}(t)$ are the control amplitude of the i channel RF pulse.

For numerical optimization, the time period of the pulse is divided into N equal slices, i.e., $\tau = N\Delta t$, and the time-dependent Hamiltonian is approximated with the piecewise constant function. The spin dynamics can be described as the density matrix $\rho(t)$ evolves under the propagator $\mathbf{P}(t)$. At the end of the pulse, the final spin state is given by[3]

$$\rho(\tau) = \mathbf{P}_N \cdots \mathbf{P}_2 \mathbf{P}_1 \rho_0 \tag{S12}$$

where the k-step propagator $\mathbf{P}_k$ is

$$\mathbf{P}_k = \exp[-i(\mathbf{H}_{\text{int,k}} + \mathbf{H}_{\text{rf,k}} + \mathbf{H}_{\text{g,k}})\Delta t] \tag{S13}$$

The fidelity function is measured by the inner product

$$\eta = < \rho_T | \rho(\tau) > = \text{trace}[\rho_{\text{T}}^{\dagger} \rho(\tau)] \tag{S14}$$

For CLOC pulse, the target state ρ_T should be consistent with the initial state. For non-Hermitian states, the fidelity takes the real value of Eq. S14. In this sense, the target

propagator $\mathbf{P}$ commutes with the initial state. For example, we have $[\mathbf{P}, I^-] = 0$ for locking of single spin state I^-, or $[\mathbf{P}, I^-S^-] = 0$ for locking of double quantum coherence I^-S^-.

In addition to the resonance offsets and RF inhomogeneity, two types of ensembles were defined in the optimal control model: the drift Hamiltonians and the source-target pairs. To account for gradient spillover, which induces a time- and space-dependent B_0 drift, the sample was divided into n_g voxels along the z direction. Each voxel was assigned a time-dependent drift Hamiltonian. The system Hamiltonian was constructed by assembling the drifts from all voxels into the blocks of a block-diagonal matrix. Regarding the source-target pairs, it's practical to specify three coherences (I_z, I^- and I^+) for a single spin, so the optimal pulse is actually a cyclic pulse. For multiple spins, including multiple coherences can be highly time-consuming, potentially requiring several days with high-performance computers.

The fidelity function in Eq. S14 was expanded to

$$\overline{\eta} = \frac{1}{n} \sum_{n_{\mathrm{rf}}} \sum_{n_{\mathrm{g}}} \sum_{n_{\mathrm{off}}} \sum_{n_{\rho}} \eta \tag{S15}$$

where n_{rf}, n_{g}, n_{off}, n_{ρ} denote the number of RF amplitudes, gradient strengths, resonance offsets, and source-target pairs, respectively.

Parameters setting The following parameters were fixed for the single spin optimization.

- Pulse duration was 1 ms, divided into 1000 equivalent intervals.
- The gradient pulse has a sine shape, and the duration was 1 ms. The gradient field was calculated at 12 voxels along the z-direction, i.e., $n_{\mathrm{g}} = 12$, the maximum value of generated B_0 drift was $B_{\mathrm{g,max}} = \pm 0.25$ Gauss.
- ^{1}H offset, bandwidth is 7 kHz, $n_{\mathrm{off}} = 51$.
- ^{1}H RF amplitude, $\nu_{1,\mathrm{H}} = 6$ kHz, with $\pm 20\%$ inhomogeneity, $n_{\mathrm{rf}} = 15$.
- ^{13}C offset, bandwidth is 6 kHz, $n_{\mathrm{off}} = 51$.
- ^{13}C RF amplitude, $\nu_{1,\mathrm{C}} = 4$ kHz, with $\pm 15\%$ inhomogeneity, $n_{\mathrm{rf}} = 10$.

Concurrent optimization is crucial when the J coupling cannot be neglected or averaged out by single spin pulses. The concurrent optimization used identical parameters but focused on a specific coherence, combining the ^{13}C ($n_{\mathrm{off,C}} = 25$) and ^{1}H ($n_{\mathrm{off,H}} = 15$) offsets while aligning the B_1 inhomogeneity ($n_{\mathrm{rf}} = 10$) across the two channels. The average J coupling was assumed to be $J_{\mathrm{HC}} = 145$ Hz. The optimization was divided into 2 steps to accelerate optimization.

- Step 1, the bandwidth of ^{1}H and ^{13}C were divided into 10 equivalent intervals, and the RF inhomogeneity was divided into 3 intervals. The optimization stopped after 500 iterations or the fidelity $\eta > 0.9985$, the calculated pulse was imported to step 2 as the initial guess.

- Step 2, the parameters indicated above were considered, the optimization stopped after 50 iterations or the fidelity $\eta > 0.995$.

The optimization was implemented with Spinach v2.8[4], in which the LBFGS-GRAPE algorithm was specified as the optimization method. The single spin optimization used 40 CPU cores with a frequency of 2.1 GHz, the calculation time was approximately 1 hour. The concurrent optimization used 64 CPU cores with a frequency of 2.6 GHz, the calculation time was approximately 2 days.

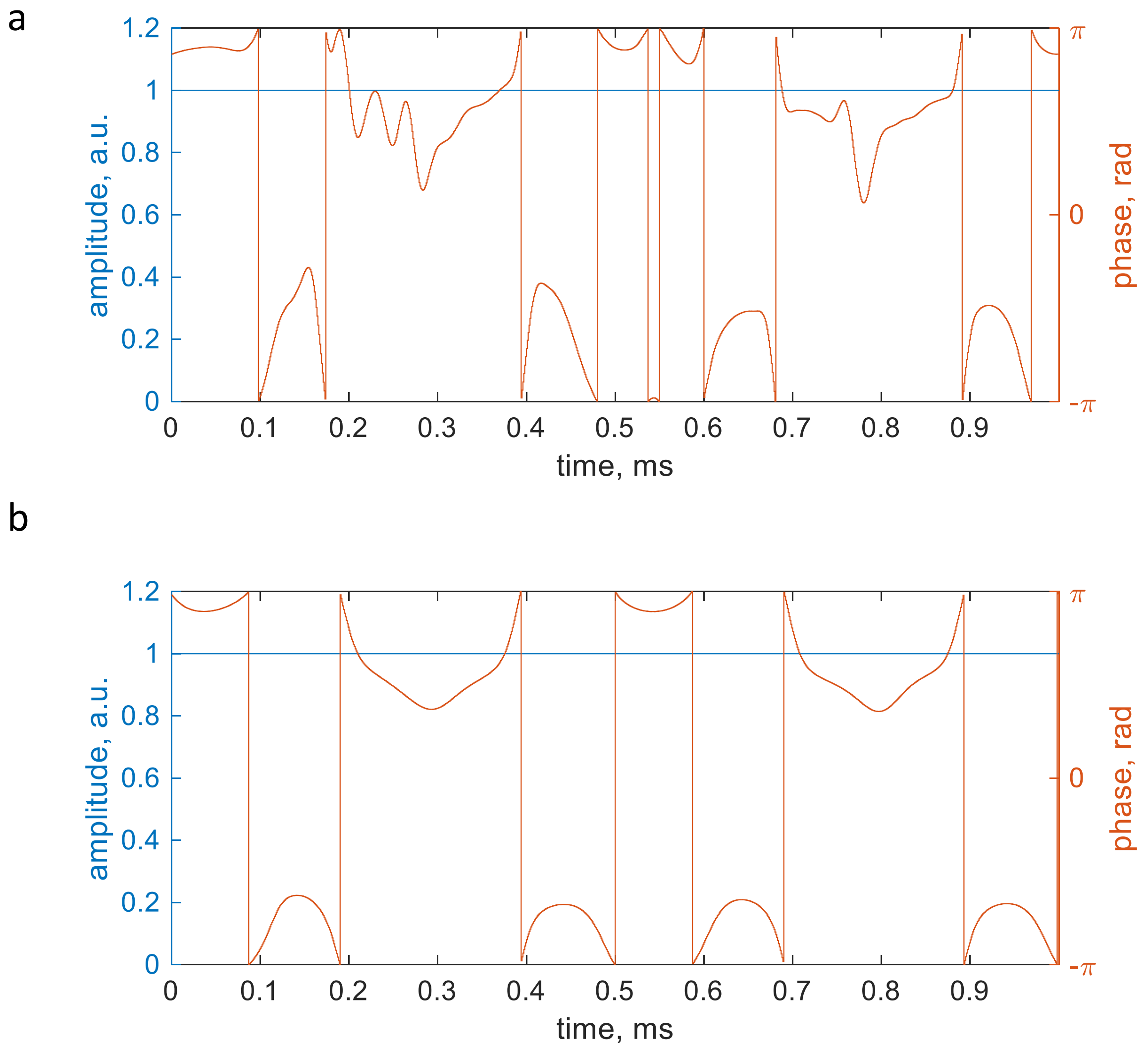

Figure S3. The CLOC pulse shapes for ^{1}H (a) and ^{13}C (b), both of which are cyclic and universally preserve the I^{-}, I^{+} and I_z.

S4. Heteronuclear decoupling and homonuclear *J*-coupling impact

The CLOC pulse has an additional heteronuclear decoupling effect due to its fast phase jump, as the noise decoupling does. Here we calculate the effective coupling scale factor using the average Hamiltonian theory[5]. In the toggling frame defined by $\mathbf{H}_{\mathrm{rf}} + \mathbf{H}_{\mathrm{z}}$, the J coupling term has the following form[6]

$$\mathbf{H}_J^{tog} = 2\pi J \sum_{\beta,\gamma} a_{z\beta}^I(t) a_{z\gamma}^S(t) I_\beta S_\gamma = 2\pi \cdot \mathbf{I} \cdot \mathbf{J} \cdot \mathbf{S} \tag{S16}$$

where $\mathbf{J}$ is a 3 by 3 time-dependent coupling tensor, its elements are given by

$$c_{\beta,\gamma}(t) = a_{z\beta}^I(t) a_{z\gamma}^S(t) \tag{S17}$$

where β, γ equal to x, y, z. The rotation coefficients $a_{z\beta}^I(t)$ can be calculated by

$$a_{z\beta}^I(t) = < \mathbf{P}_I^\dagger(t) I_z | I_\beta > \tag{S18}$$

where the propagator $\mathbf{P}_I$ concerning the single spin I is defined by

$$\mathbf{P}_I(t) = T \exp\left[-\mathrm{i} \int_0^t \mathbf{H}_I(t')\, dt'\right] \tag{S19}$$

where T is the Dyson time-ordering operator, the single spin Hamiltonian is given by

$$\mathbf{H}_I(t) = \mathbf{H}_{I,\mathrm{z}} + \mathbf{H}_{I,\mathrm{rf}} = \omega_I I_z + \omega_{I,x}(t) I_x + \omega_{I,y}(t) I_y \tag{S20}$$

The calculated time-dependent coupling tensor in Eq. S17 was averaged over the pulse period τ, i.e.,

$$\overline{c_{\beta,\gamma}} = \frac{1}{\tau} \int_0^\tau c_{\beta,\gamma}(t)\, dt \tag{S21}$$

The coupling scale factor is defined as the Frobenius norm of averaged coupling tensor since the maximum value of its norm is 1, i.e.,

$$\chi = \mathrm{norm}(\bar{\mathbf{c}}) \tag{S22}$$

Due to homonuclear coupling, we observed intensity loss in the glucose HSQC spectra when coherence-locking pulses were applied. This homonuclear coupling can be compensated in optimal control. Specifically, when optimizing a universal locking pulse for a single ^{13}C spin, the model includes two ^{13}C spins, the resonance offset is specified for only one spin, while the other is fixed at zero offset. A J-coupling Hamiltonian,

$$\mathbf{H}_{\mathrm{J}} = 2\pi J (I_{1x} I_{2x} + I_{1y} I_{2y} + I_{1z} I_{2z}) \tag{S23}$$

is included in the drift Hamiltonian, where J is set to a representative value, such as 40 Hz for J_{CC}. The compensation increases the RF power required for the optimal control pulse.

However, when two spins in a general spin system have a chemical shift difference comparable to the J-coupling constant, compensation becomes infeasible. Since $[\mathbf{H}_{rf}, \mathbf{H}_J] = 0$ and $[\mathbf{H}_z, \mathbf{H}_J]$ is nearly zero, $\mathbf{H}_J$ cannot be averaged out in the toggling frame defined by $\mathbf{H}_{rf} + \mathbf{H}_z$. Consequently, the second-order spectrum cannot be fully recovered through coherence locking. Fig. S4 shows the impact of homonuclear coupling on the locking efficiency of the ^{13}C CLOC pulse.

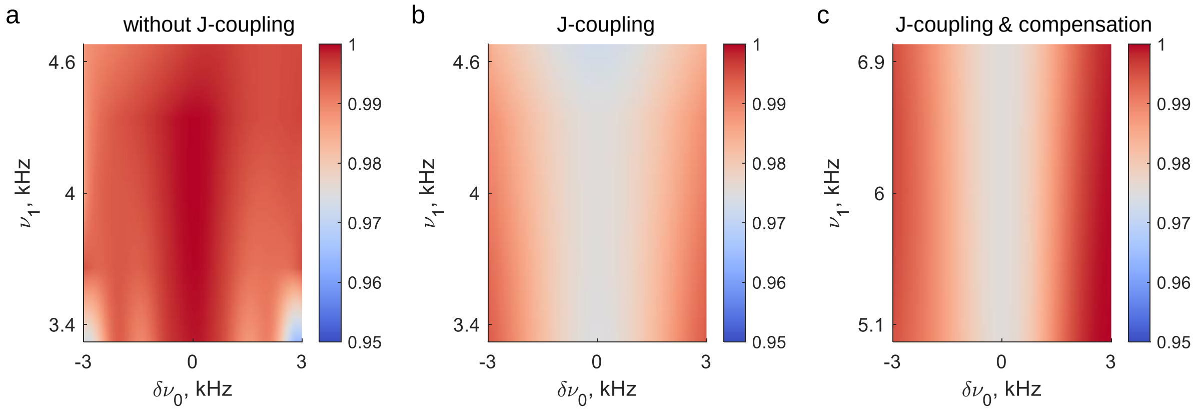

Figure S4. Simulated coherence locking efficiency of the ^{13}C CLOC pulse subject to a two-^{13}C spin system. The efficiency is defined as $\eta = <\rho_0|\rho_T>$, where ρ_0 is the initial state, ρ_T is the final state. The $\delta v_0 = \omega_1 - \omega_2$, where ω_1 is fixed at 0, and ω_2 is swept from -3 kHz to 3 kHz, $J = 50$ Hz, and v_1 is the RF amplitude of the pulse. (a) $\rho_0 = I_{1x} + I_{2x}$, which commutes with $\mathbf{H}_J$. (b-c) $\rho_0 = I_{1x}$, using the CLOC pulse without J-coupling compensation (b) and with J-coupling compensation, where the RF amplitude is increased to 6 kHz (c).

S5. Simulation of coherence-locking efficiency

The coherence-locking efficiency as a function of resonance offset, RF inhomogeneity, and gradient field strength were provided in Fig. S5 and Fig. S6. The efficiency was calculated by the following fidelity function, where **E** is an identity matrix indicating the target propagator, and **P** is the actual propagator.

$$\eta = \mathrm{trace}(\mathbf{E}^{\dagger}\mathbf{P}) \tag{S24}$$

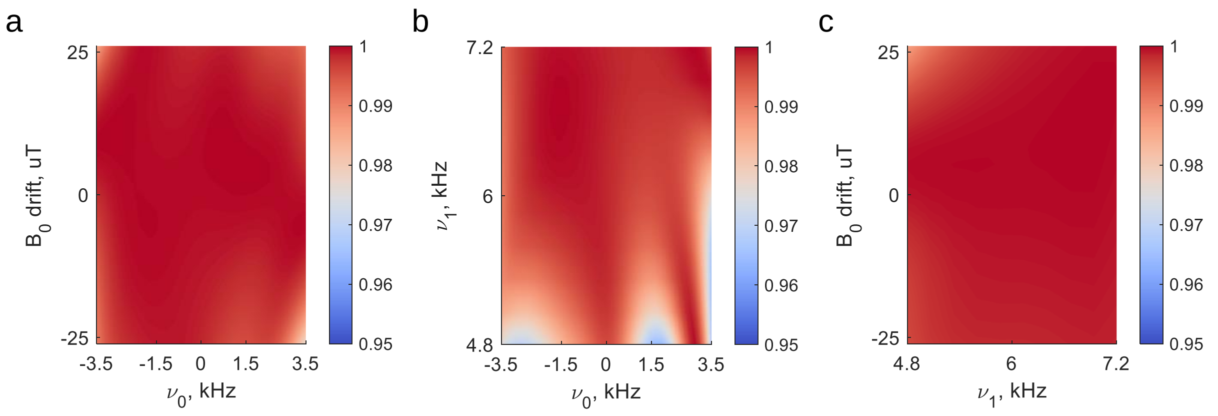

Figure S5. Efficiency of ^{1}H CLOC pulse. (a) $\nu_1 = 6$ kHz. (b) The B_0 drift is 0.25 Gauss. (c) $\nu_0 = 0$.

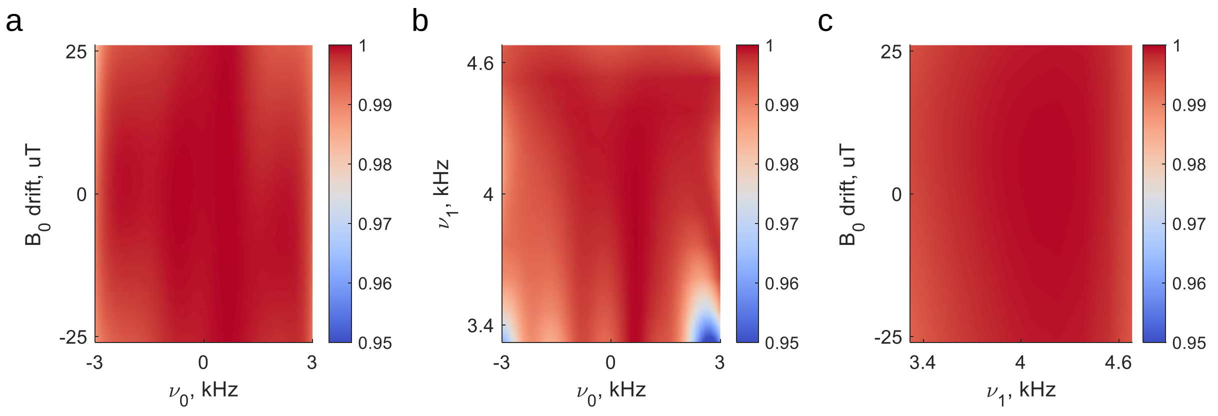

Figure S6. Efficiency of ^{13}C CLOC pulse. (a) $\nu_1 = 4$ kHz. (b) The B_0 drift is 0.25 Gauss. (c) $\nu_0 = 0$.

S6. Simulation of parallel HSQC

parameters	glycine (channel 1)		glucose (channel 2)	
gradient ratio	4:1		4:-1	
	^{1}H	^{13}C	^{1}H	^{13}C
sweeping frequency, Hz	2k	5k	2k	6.25k
transmit offset, Hz	2k	5k	2k	10.625k
sampling points	128	128	128	128
zero-fill points	512	512	512	512

Table S1 The parameters for simulating parallel HSQC spectra. The spin system of glucose utilized literature data for α-D-glucopyranose in D_2O (25 °C, pH 7.0)[7], the ^{13}C-^{13}C J-coupling was ignored. For each t1 point, both S^+ and S^- were recorded by flipping the second gradient pulse in each channel, resulting in a quadrature signal. The signal was apodized using the $\cos^2(\mathrm{x})$ half-bell function in both dimensions, and the real part of the Fourier transform was shown. A homogeneous B_0 field was assumed ($B_0 = 11.74$ T). The sample was divided into 400 voxels along the z-direction, maximum B_0 drift by primary gradient was ±25 G, and maximum B_0 drift by coupled gradient was ±0.2 G. A homogeneous B_1 field was assumed for CLOC pulses, perfect excitation and inversion pulses were assumed. $J = 145$ Hz.

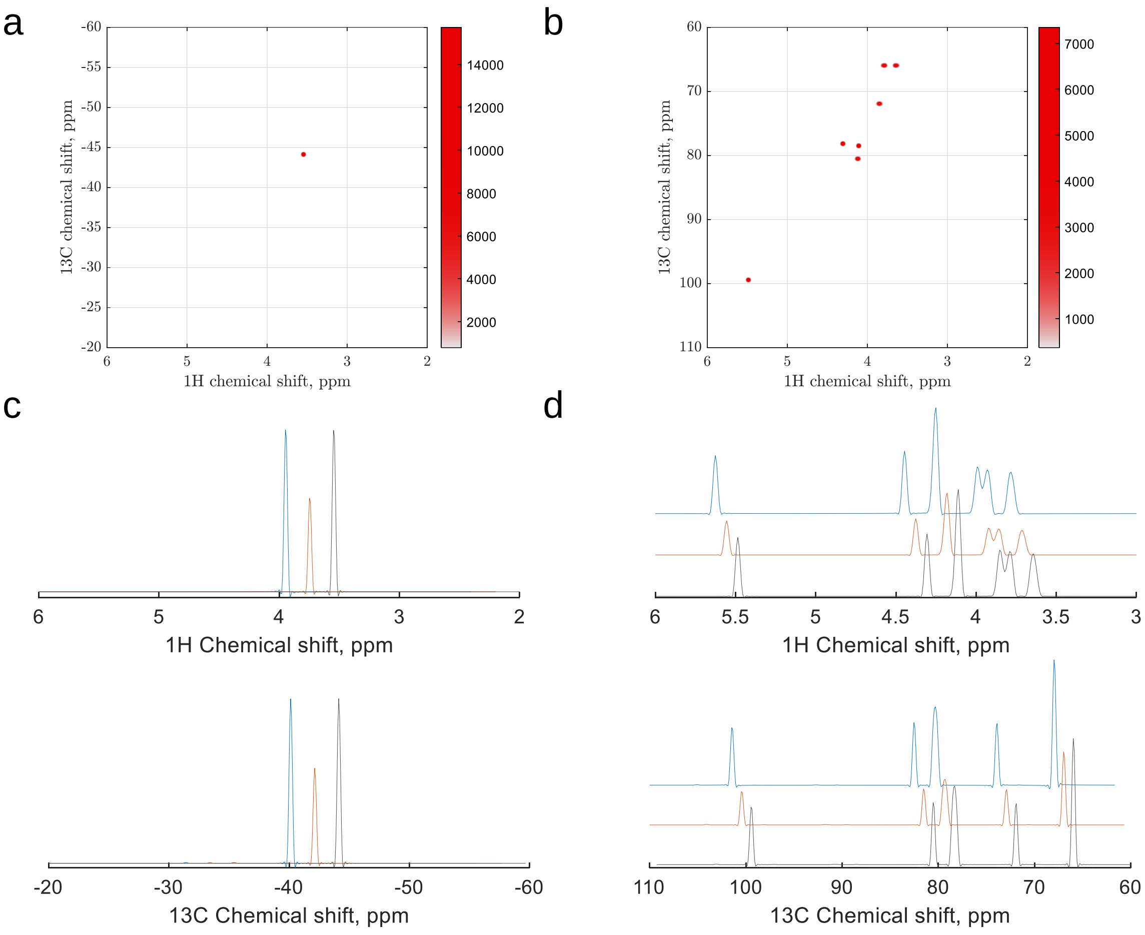

Figure S7. Simulated parallel HSQC spectra of glycine (a) and glucose (b). Panels (c) and (d) present the corresponding 1D projections of (a) and (b), respectively. The grey lines represent the results without gradient coupling, as a reference. The orange 1D projections display the spectra under gradient spillover (no compensation), and the blue 1D projections display the results with coherence-locking compensation, the lines are shifted just for clarity. Note that I^+S^+ and I^-S^+ were involved in the pathway for glycine, hence the ^{13}C signal picks up the $-\omega_C$, representing the negative chemical shifts.

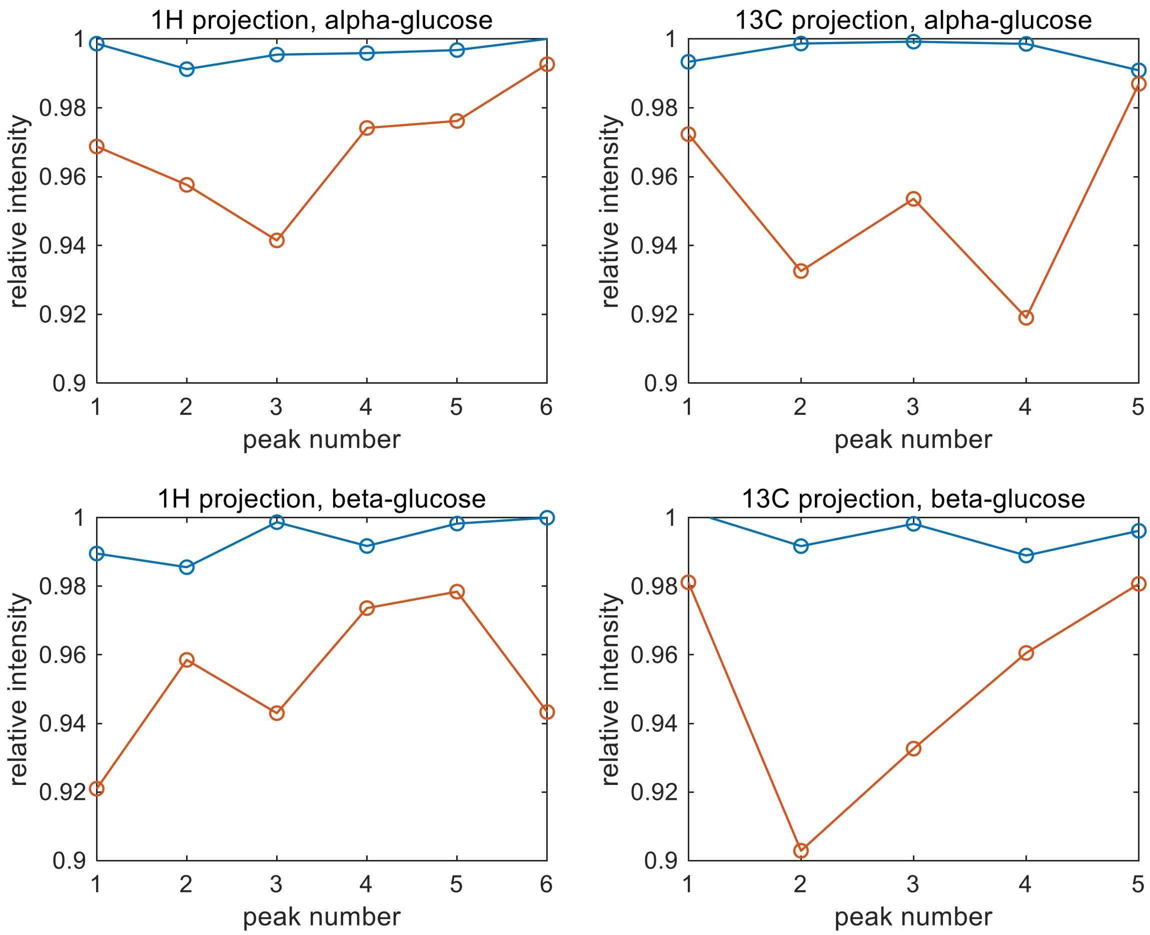

Figure S8. Simulated strong coupling effects on the glucose HSQC spectra. The relative intensity represents the ratio of peak values in the coherence-locking case to those in the standard case. The simulation excluded (blue lines) and included (orange lines) homonuclear J-coupling. The peak missing is due to insufficient spectral resolution. The spin systems were taken form literature[8–10].

S7. Simulation of parallel HMQC

The coherence protection scheme relies on optimizing the CLOC pulse for a particular coherence. A similar compensation approach was employed in the simulation of parallel HMQC experiments[2], as depicted in Fig. S9. In detector 1, the gradient pulse ratio was configured as 2:2:1 to select the $I^+S^+ \rightarrow I^-S^+ \rightarrow I^-$ pathway. Conversely, in detector 2, the gradient pulse ratio was adjusted to 2:2:-1 to select the $I^+S^- \rightarrow I^-S^- \rightarrow I^-$ pathway. The CLOC pulses in the HMQC were applied to the ^{1}H & ^{13}C channel, dependent on the double quantum coherence to be protected. The simulated spectra of the parallel HMQC pulse sequence are presented in Fig. S10, with glycine and glucose again serving as the samples in the respective detectors.

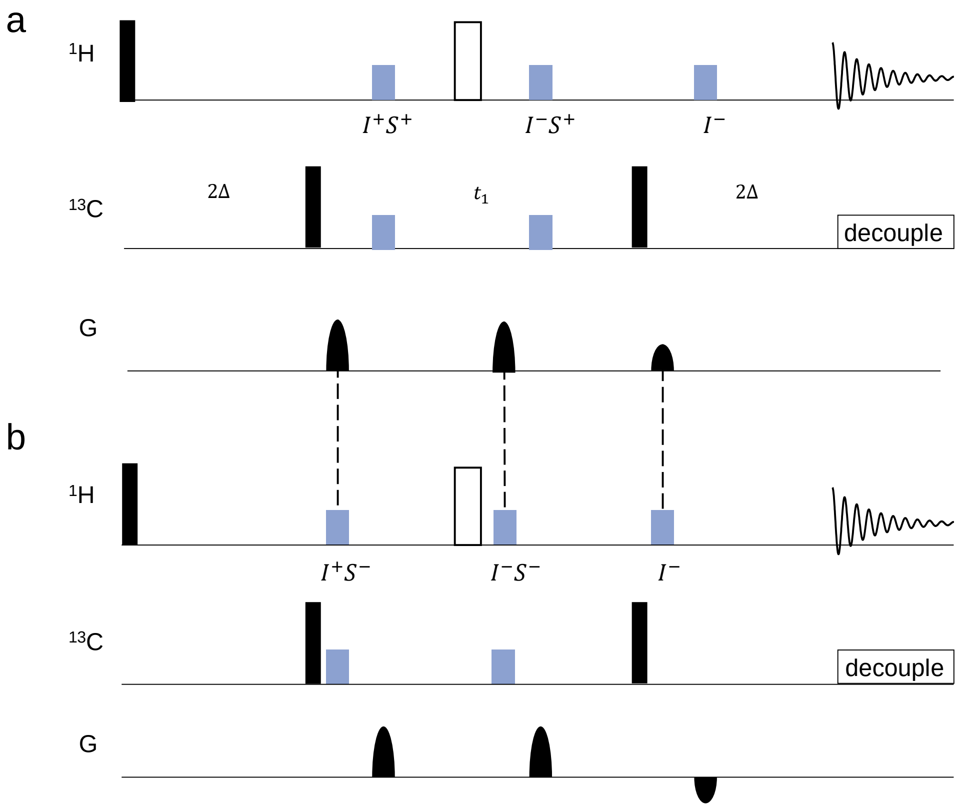

Figure S9. The scheme for gradient pulse compensation in a parallel HMQC pulse sequence involves coherence locking indicated by the blue blocks. In 1 (a), the gradient pulse ratio was set to 2:2:1 to select the $I^+S^+ \rightarrow I^-S^+ \rightarrow I^-$ pathway. In 2 (b), the gradient pulse ratio was set to 2:2:-1 to select the $I^+S^- \rightarrow I^-S^- \rightarrow I^-$ pathway. The black blocks represent $\pi/2$ pulses and the white blocks represent π pulses, with all phases set to zero unless specifically noted. $\Delta = 1/4J$.

parameters	glycine (channel 1)		glucose (channel 2)	
gradient ratio	2:2:1		2:2:-1	
	^{1}H	^{13}C	^{1}H	^{13}C
sweeping frequency, Hz	2k	5k	2k	6.25k
transmit offset, Hz	2k	5k	2k	10.625k
sampling points	256	256	256	256
zero-fill points	512	512	512	512

Table S2 The parameters for simulating parallel HMQC spectra. Only S^{+} or S^{-} was recorded for each channel, the signal was apodized using the cos(x) half-bell function in both dimensions, and the magnitude of the Fourier transform was shown. A homogeneous B_0 field was assumed ($B_0 = 11.74$ T). The sample was divided into 400 voxels along the z-direction, maximum B_0 drift by primary gradient was ±25 G, and maximum B_0 drift by coupled gradient was ±0.2 G. The B_1 field for CLOC pulse was linearly scaled according to the voxel position, i.e., maximum at the center and minimum at the edges. Perfect excitation and inversion pulses were assumed. $J = 145$ Hz.

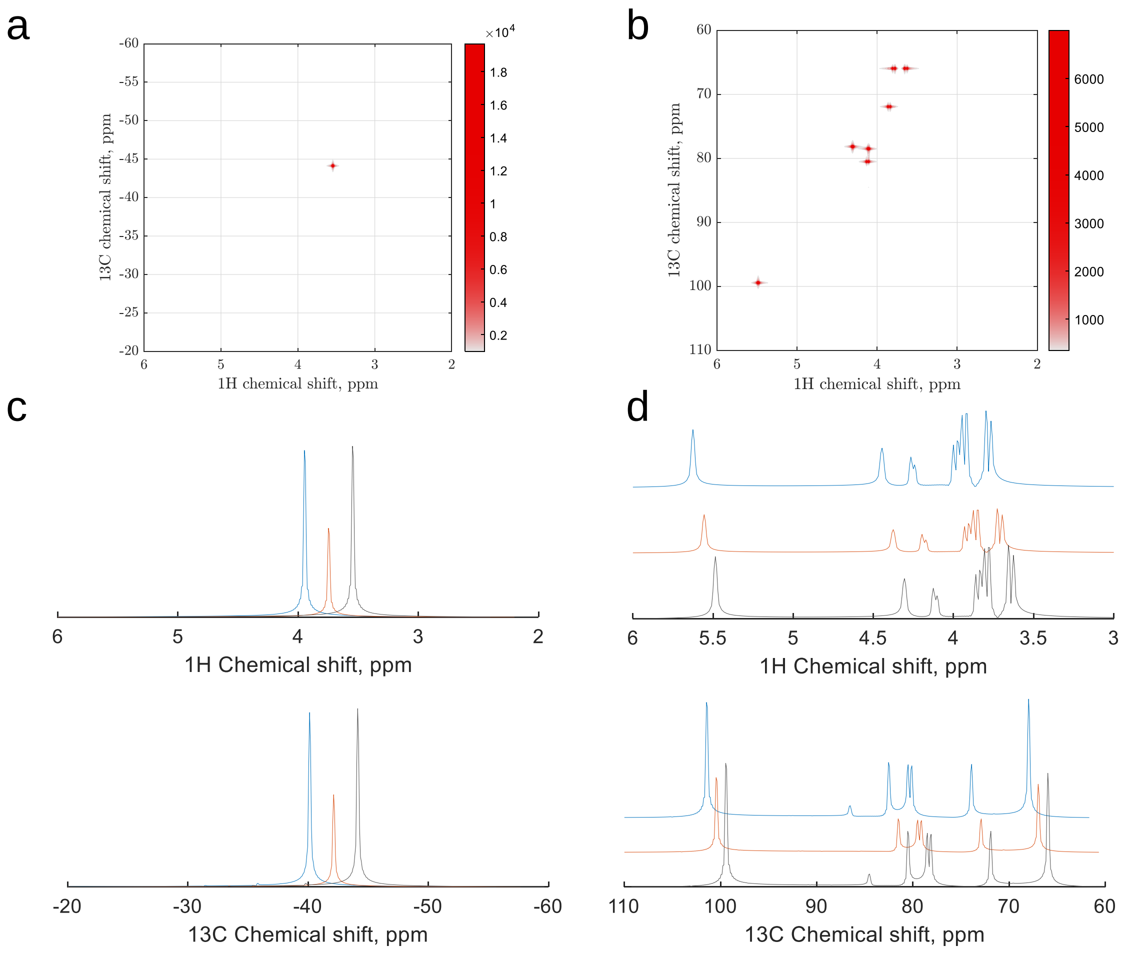

Figure S10. Simulated parallel HMQC spectra of glycine (a) and glucose (b). Panels (c) and (d) present the corresponding 1D projections of (a) and (b), respectively. The grey lines represent the results without gradient coupling, as a reference. The orange 1D projections display the spectra under gradient spillover (no compensation), and the blue 1D projections display the results with coherence-locking compensation, the lines are shifted just for clarity. In (d), the small peaks in the middle of the ^{13}C projection indicate a zero-frequency component. The averaged J-coupling evolution time causes imperfect coherence transfer, leaving a residual transfer ($I^+ \to I^- \to I^-$) that was not fully averaged out.

S8. Influence of gradient imperfection

The gradient coil can be modeled as a resistor–inductor circuit, which processes low-pass character, resulting in smoothing and phase delay of the gradient pulse. These transient effects change the temporal alignment with the RF pulse and can cause efficiency degradation of coherence locking. Here we gave the simulated locking performance considering the gradient imperfection.

The quality factor of the gradient coil is $Q = \omega L/R$ where $\omega = \pi/\tau$ and τ is the duration of the 'sine' shape pulse. The phase delay of the circuit is $\phi = \tan^{-1}(Q)$. The coherence-locking efficiency considering phase delay response is given in Fig. S11.

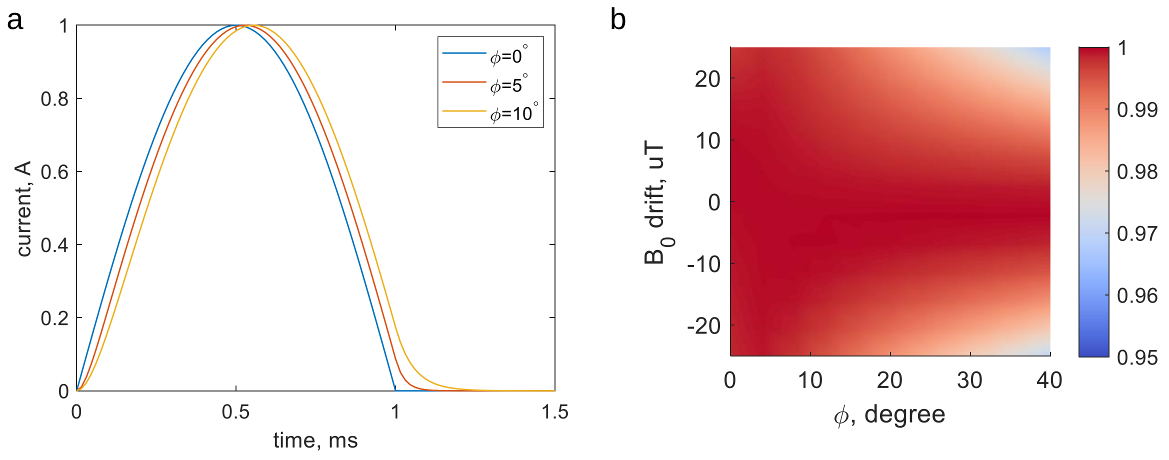

Figure S11. Coherence-locking efficiency considering the transient response of gradient coil. (a) The transient response of gradient coil at different delay phases. (b) Coherence-locking efficiency of I^- as a function of delay phase and B_0 drift, calculated using the ^{1}H CLOC pulse.

S9. The parallel probe and parallel HSQC spectra

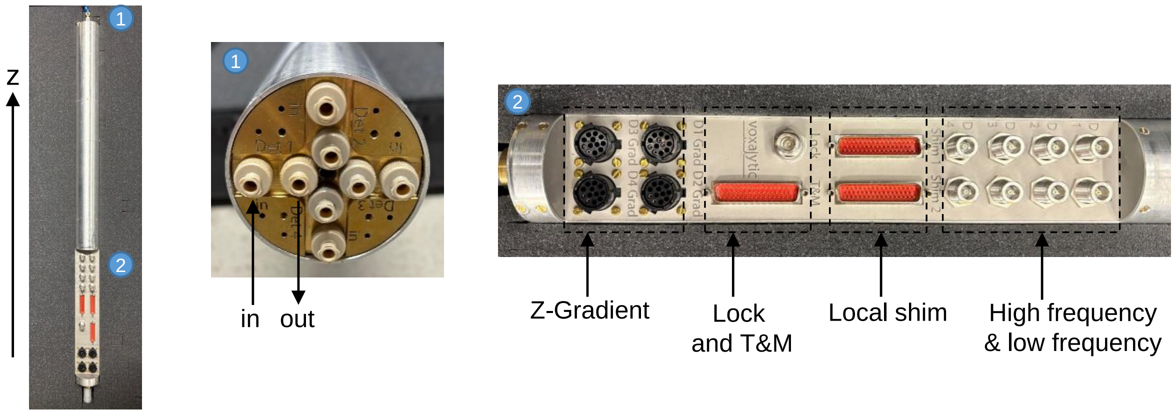

Figure S12. View of the 4-detector probe (Voxalytic GmbH).

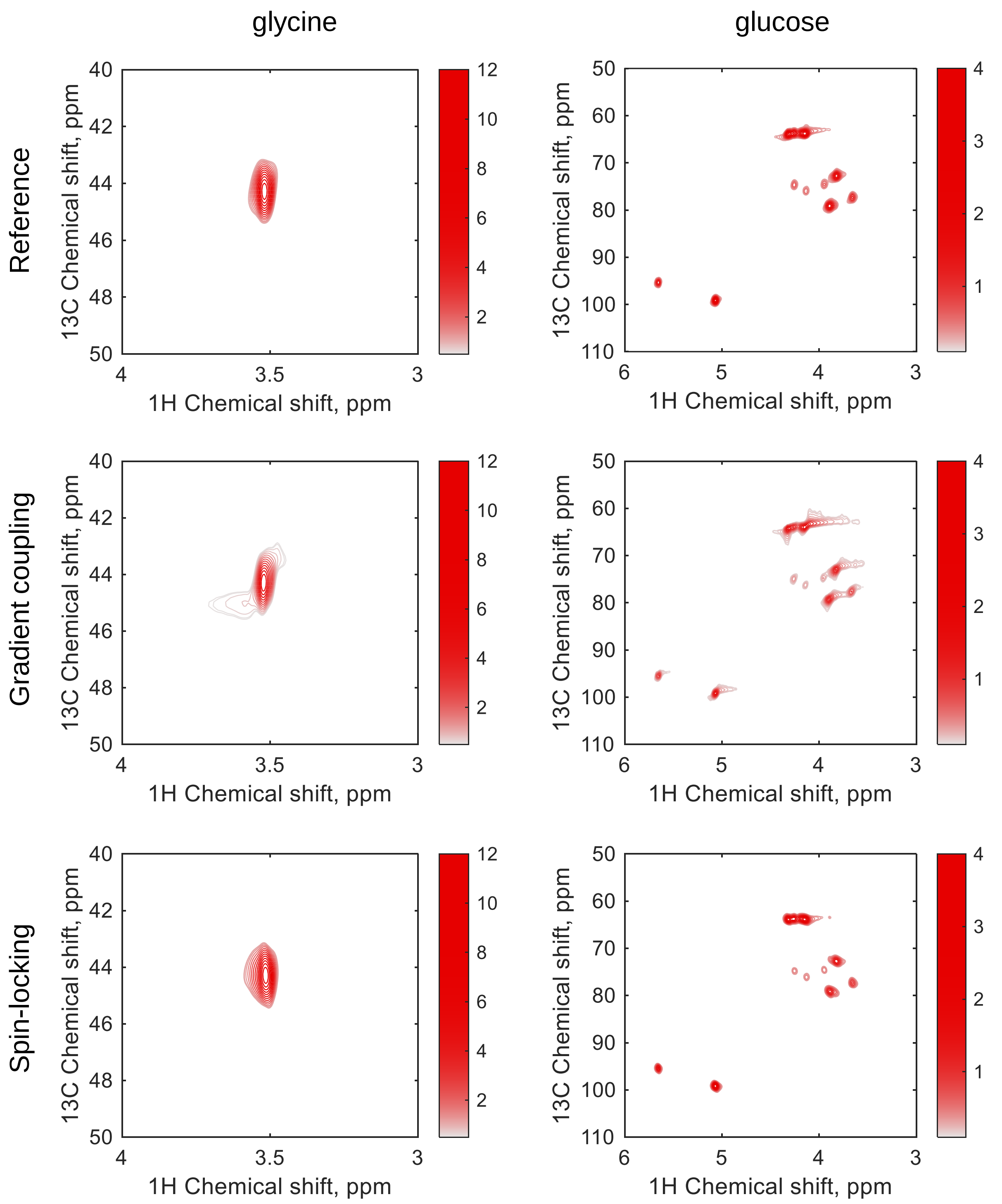

Figure S13. Parallel HSQC spectra. Top panels: the spectra without gradient coupling. Middle panels: the spectra under gradient spillover. Bottom panels: the spectra with coherence-locking compensation. The 2D spectra were saved from TopSpin and plotted with Matlab using a Spinach utility function `'plot_2d.m'`.

Supplementary References

1. M. H. Levitt, *Spin Dynamics: Basics of Nuclear Magnetic Resonance* (John-Wiely, 2008), pp. 649–652.

2. J. Ruiz-Cabello, G. W. Vuister, C. T. W. Moonen, P. van Gelderen, J. S. Cohen, P. C. M. van Zijl, Gradient-enhanced heteronuclear correlation spectroscopy. theory and experimental aspects. *J. Magn. Reson.* **100**, 282–302 (1992).

3. N. Khaneja, T. Reiss, C. Kehlet, T. Schulte-Herbruggen, S. J. Glaser, Optimal control of coupled spin dynamics: Design of NMR pulse sequences by gradient ascent algorithms. *J. Magn. Reson.* **172**, 296–305 (2005).

4. H. J. Hogben, M. Krzystyniak, G. T. Charnock, P. J. Hore, I. Kuprov, Spinach–a software library for simulation of spin dynamics in large spin systems. *J. Magn. Reson.* **208**, 179–194 (2011).

5. J. S. Waugh, Theory of broadband spin decoupling. *J. Magn. Reson.* **50**, 30–49 (1982).

6. S. J. Glaser, J. J. Quant, *Homonuclear and Heteronuclear Hartmann—Hahn Transfer in Isotropic Liquids* (Academic Press, 1996), vol. 19, pp. 59–254e.

7. E. Alexandersson, G. Nestor, Complete 1H and 13C NMR spectral assignment of d-glucofuranose. *Carbohydr. Res.* **511**, 108477 (2022).

8. K. Kamieńska-Trela, One-bond 13C-13C spin-spin coupling constants. *Annual Reports on NMR Spectroscopy*, G. A. Webb, ed. (Academic Press, 1995), vol. 30, pp. 131–230.

9. M. U. Roslund, P. Tähtinen, M. Niemitz, R. Sjöholm, Complete assignments of the 1H and 13C chemical shifts and *J*H,H coupling constants in NMR spectra of D-glucopyranose and all D-glucopyranosyl-D-glucopyranosides. *Carbohydr. Res.* **343**, 101–112 (2008).

10. B. Yu, H. van Ingen, S. Vivekanandan, C. Rademacher, S. E. Norris, D. I. Freedberg, More accurate 1*J*CH coupling measurement in the presence of 3*J*HH strong coupling in natural abundance. *J. Magn. Reson.* **215**, 10–22 (2012).
